# Supplementary material for: Comparative transcriptomic analysis of perfluoroalkyl substances-induced responses of exponential and stationary phase Escherichia coli
Source: BMC Genomics. 2025 Nov 7;26:1016. doi: 10.1186/s12864-025-12109-4 (PMC12595759; doi:10.1186/s12864-025-12109-4)
Supplement: Supplementary file 4 — Supplementary Material 4. [file 12864_2025_12109_MOESM4_ESM.pdf]

Supplementary Note 1. Two-way mixed ANOVA for growth data. A two-way mixed ANOVA of optical density measurements and time was performed in RStudio running R version 4.2.2 with functions from packages tidyverse (v2.0.0) and rstatix (v0.7.2). The R script used to perform this analysis is provided below. Input and output data tables are provided in SI Table 1.

The between-subjects factors are the PFCA and NFCA samples (treatment) and the within-subjects factor is time. We assumed no significant outliers were present in the dataset, normality, and homogeneity of variances. We used Levene's test for equality of variances to assume sphericity and homogeneity of covariances.

Key R functions used in the analysis: anova_test( ), levene_test( ), get_anova_table( ) from rstatix.

R script:

```
# Mixed two-way ANOVA - E. coli seq dataset


# Load required libraries

library(tidyverse)

library(ggpubr)

library(rstatix)

# Load in OD dataframe (SI Table 1)

od_data_ec <- read.delim("/path/input_anova.txt")

# Gather columns into long format (group times in single column)

# Convert 'id' and 'time' into factor variables

od_data_ec <- od_data_ec %>%

  gather(key = "time", value = "score", t0, t2, t4, t6, t24, t48) %>%

  convert_as_factor(id, time, group)

# Visualize long-form to make sure it is correct

set.seed(123)

od_data_ec %>% sample_n_by(group, time, size = 1)
```

```
# Perform summary stats
# Output table provides: mean, sd, count
od_data_ec %>%
  group_by(time, group) %>%
  get_summary_stats(score, type = "mean_sd")
# Plot
bxp <- ggboxplot(
  od_data_ec, x = "time", y = "score",
  color = "group", palette = "jco"
)
bxp
# Check out assumptions for ANOVA:
od_data_ec %>%
  group_by(time, group) %>%
  identify_outliers(score)

# QQ plot draws the correlation between a given data and the normal distribution
# Points fall along the line for normal distribution
ggqqplot(od_data_ec, "score", ggtheme = theme_bw()) +
  facet_grid(time ~ group)

# Homogeneity of variance assumption
# The homogeneity of variance assumption of the between-subject factor (group)...
# ...can be checked using the Levene's test
od_data_ec %>%
  group_by(time) %>%
  levene_test(score ~ group)
```

```
# Homogeneity of covariances assumption
# Box's M-test
# If this test is statistically significant (i.e., p < 0.001), you do not have
# equal covariances, but if the test is not statistically significant
# (i.e., p > 0.001), you have equal covariances and you have not violated the
# assumption of homogeneity of covariances.
box_m(od_data_ec[, "score", drop = FALSE], od_data_ec$group)

#----Run ANOVA-----
# Two-way mixed ANOVA test
res.aov.ec <- anova_test(
  data = od_data_ec, dv = score, wid = id,
  between = group, within = time
)
res_anova_table_ec <- get_anova_table(res.aov.ec)

#-------- Post-hoc tests----------
# Simple main effect of group variable - investigate effect of b/w subject ...
# ...factor (group-condition) on OD600 (score) at every time point
# Impact of group at each time point
one.way.ec <- od_data_ec %>%
  group_by(time) %>%
  anova_test(dv = score, wid = id, between = group) %>%
  get_anova_table() %>%
  adjust_pvalue(method = "bonferroni")
one.way.ec
```

```
# Pairwise comparisons between group levels
pwc_ec <- od_data_ec %>%
  group_by(time) %>%
  pairwise_t_test(score ~ group, p.adjust.method = "bonferroni")
pwc_ec

pwc_ec <- pwc_ec %>% add_xy_position(x = "time")

bxp +
  stat_pvalue_manual(pwc.filtered, tip.length = 0, hide.ns = TRUE) +
  labs(
    subtitle = get_test_label(res.aov.pp, detailed = TRUE),
    caption = get_pwc_label(pwc_pp)
  )

# Coerce the data.frame to all-character
df_out = data.frame(lapply(pwc_ec, as.character), stringsAsFactors=FALSE)

# write out results table
write.csv(df_out, "/path/anova_output.csv")
```
